# Development of Electrochemical Immunosensors for HER-1 and HER-2 Analysis in Serum for Breast Cancer Patients

**DOI:** 10.3390/bios13030355

**Published:** 2023-03-07

**Authors:** Shayalini Wignarajah, Iva Chianella, Ibtisam E. Tothill

**Affiliations:** School of Aerospace, Transport and Manufacturing, Cranfield University, Cranfield, Bedfordshire MK43 0AL, UK

**Keywords:** electrochemical immunosensor, HER-1 or EGFR, HER-2, serum, amperometry, gold sensor chips, breast cancer, gold nanoparticle

## Abstract

In this work, two human epidermal growth factor receptors, HER-1 and HER-2, were selected as biomarkers to enable the detection of breast cancer. Therefore, two biosensors were developed using gold sensor chips coupled with amperometric detection of the enzyme label horse radish peroxidase (HRP). The biosensors/immunosensors relied on indirect sandwich enzyme-linked immunosorbent assays with monoclonal antibodies (Ab) against HER-1 and HER-2 attached to the sensors to capture the biomarkers. Detection polyclonal antibodies followed by secondary anti-rabbit (for HER-1) and anti-goat (for HER-2) IgG antibody-HRP were then applied for signal generation. In buffer, the developed sensors showed limits of detections (LOD) of 1.06 ng mL^−1^ and 0.95 ng mL^−1^ and limits of quantification (LOQ) of 2.1 ng mL^−1^ and 1.5 ng mL^−1^ for HER-1 and HER-2, respectively. In 100% (undiluted) serum, LODs of 1.2 ng mL^−1^ and 1.47 ng mL^−1^ and LOQs of 1.5 ng mL^−1^ and 2.1 ng mL^−1^ were obtained for HER-1 and HER-2, respectively. Such limits of detections are within the serum clinical range for the two biomarkers. Furthermore, gold nanoparticles (AuNP) labelled with secondary anti-rabbit and anti-goat IgG antibody-HRP were then used to enhance the assay signal and increase the sensitivity. In buffers, LODs of 30 pg mL^−1^ were seen for both sensors and LOQs of 98 pg mL^−1^ and 35 pg mL^−1^ were recorded for HER-1 and HER-2, respectively. For HER-2 the AuNPs biosensor was also tested in 100% serum obtaining a LOD of 50 pg mL^−1^ and a LOQ of 80 pg mL^−1^. The HER-2 AuNP electrochemical immunosensor showed high specificity with very low cross-reactivity to HER-1. These findings demonstrate that the two developed sensors can enable early detection as well as monitoring of disease progression with a beneficial impact on patient survival and clinical outcomes.

## 1. Introduction

Breast cancer is a type of cancer that affects the human breast. It is the most frequently diagnosed type of cancer and is the leading cause of death by cancer in women both in the UK and worldwide. For example, in 2018, there were more than 2 million newly diagnosed breast cancer cases worldwide with 629,679 total deaths [1,2]. In the UK, the number of people diagnosed with breast cancer between 2016 and 2018 was 55,920 with 11,547 deaths [3]. The risk of breast cancer increases with age, gender, gene mutations and family history. Cancer can also be classified depending on the affected tissue of the breast, which can be differentiated by anatomic site, distribution and whether the cancer is invasive or non-invasive [4]. Accurate clinical staging for breast cancer has been used as a guide to both prognosis and treatment with five stages based on the size and spread of the tumor [4].

Currently, there are various methods used in hospitals to diagnose and detect breast cancer. The most utilized methods are self-examination, clinical breast examination, mammography and biopsy, which are paramount for the early detection of the tumor [4]. Breast imaging (mammography) [5], together with blood tests (e.g., general blood count for tumor identification) are required for the diagnosis and monitoring of breast cancer [4]. However, mammography has its limitations and can be painful. Hence, research on blood biomarkers has expanded with breakthroughs in identifying specific markers, which can be used in the prediction and prognosis of the disease. To date, there are no specific breast cancer biomarkers that can be used for the early detection of cancer. Nonetheless, a few are being considered in clinical routines for prevention, prognosis, prediction, and monitoring of treatment [6]. In fact, a few biomarkers found in breast cancer patients show different concentrations depending on the stage of the disease [7].

In this work, two breast cancer biomarkers were selected to develop sensors for cancer diagnosis, and these are the human epidermal growth factor receptors 1 and 2 (HER-1 and HER-2). Since the overexpression of HER-1 and HER-2 can cause aggressive tumor behavior, they can be used to predict and monitor the progression in metastatic situations. Both HER-1 and HER-2 belong to the human epidermal growth factor receptor family which includes, HER-1 (EGFR), HER-2 (erbB2), HER-3 (erbB3), and HER-4 (erbB4) [8]. HER-1 (or EGFR) is also a member of the transmembrane receptor family [9] and is a vital mediator of cancer cell transformation, proliferation, survival, adhesion, migration, maintenance, and differentiation [10]. Overexpression of HER-1 has been linked to uncontrolled tumor proliferation and apoptosis [9,11] as well as poor survival and poor response to therapy [10], which makes it a good biomarker to indicate disease progression and response to treatment. HER-1 is overexpressed in 14–90% of breast cancers, depending on the sample type tested (e.g., serum or tissue) and the technique used to quantitate the receptor. The conventional standard techniques for measuring HER-1 include immunohistochemistry (IHC) on tissues, Western blotting (WB) on membranes and enzyme-linked immunosorbent assay (ELISA), which is the gold standard method [12]. All these methods can have high sensitivity and selectivity, but are time-consuming, expensive and require skilled personnel [12].

HER-2 is the only growth factor receptor out of the existing four that is permanently in an activated state (homodimerization) and contains no known direct activating ligand [7]. It has been linked to cell proliferation, survival, differentiation, angiogenesis, and invasion [7,10]. HER-2 is required in breast development for lobular differentiation and milk production and its protein and corresponding gene are said to be over-expressed in breast cancer. HER-2 overexpression has been reported in 20–25% of all breast cancers [13]. However, as well as testing the HER-2 status in a tumor, HER-2 can also be detected in serum (sHER-2), hence, presenting itself as a potential blood/serum biomarker for the detection of breast cancer. HER-2 is being used in hospitals and laboratories for targeted therapy, to predict and monitor the progression in metastatic situations [14,15,16,17,18]. The ELISA sHER-2 test has been cleared and approved by the food and drug administration (FDA) for clinical use with a cut-off value of ≥15 ng mL^−1^. ELISA tests have also been used in combination with immunohistochemistry (IHC) and fluorescence in situ hybridization (FISH) in determining the HER-2 status in tissue samples [16,17].

Recently, there have been several biosensing methods developed for the detection of HER-1 and HER-2 [19,20]. Biosensors for HER-2 based on surface plasmon resonance [21] and surface acoustic waves [22] have been described. However, electrochemical sensors have been the most popular for biomarkers detection due to their highly desirable analytical features such as sensitivity, selectivity, ease of use and low cost [23]. In fact, producing a sensor that is easy to fabricate in large quantities, is inexpensive and reproducible is essential for low-cost diagnostics [24,25]. Numerous examples of electrochemical biosensors particularly for HER-2, but also HER-1 have been described in the literature and these include biosensors based on voltammetry [26,27], capacitance [28,29], anodic stripping differential pulse voltammetry [30], electrochemical impedance spectroscopy [31,32,33], cyclic voltammetry [27], amperometry [34,35], linear sweep voltammetry [36], square wave stripping voltammetry [37], and chronocoulometry [38]. Unlike other techniques, electrochemical methods are less affected by sample impurities, are low cost and the equipment required is relatively simple, with electrochemical immunosensors being the most specific for cancer biomarkers detection [24].

Nanomaterials such as gold and silver nanoparticles, carbon nanotubes and quantum dots have been used to improve sensors performance [39,40,41]. Gold nanoparticles (AuNPs) have been applied in many applications due to their strong capability to adsorb biomolecules, such as protein and DNA without the loss of biological activity [39,42,43]. Furthermore, they aid in the amplification of the signal by adsorbing several enzyme molecules on the surface, thus, the signal created per bound antibody is amplified compared to the conventional assay format.

In this manuscript, electrochemical immunosensors were developed for breast cancer detection/monitoring using HER-1 and HER-2 as the targeted biomarkers. Screen-printed gold electrodes (SPGE) were applied to prepare the biosensors as they are cost-effective and easy to use for on-site detection. Indirect sandwich ELISA formats were developed and optimized achieving biosensors with high sensitivity (in the relevant clinical ranges) and high reproducibility for the two biomarkers in both buffer and commercial serum samples with and without signal amplification using AuNPs.

## 2. Materials and Methods

### 2.1. Materials and Equipment

Albumin from bovine serum (BSA), 3,3′5,5′-tetramethyl benzidine dihydrochloride hydrate (TMB), hydrogen peroxide, potassium chloride (1 M), phosphate citrate buffer tablets (0.05 M, pH 5), sodium carbonate–bicarbonate buffer tablets (0.05 M, pH 9.6), phosphate-buffered saline (PBS) tablets (0.01 M, pH 7.4), Tween 20, gold nanoparticle (40 nm), and human serum were purchased from Sigma Aldrich UK (Merck Life Science UK Limited, Gillingham, Dorset, UK). The KPL milk protein concentrate (milk concentrate) was purchased from Seracare (LGC Seracare, Milford, UK). The mouse monoclonal antibody and the rabbit polyclonal antibody against HER-1 were purchased from Sigma Aldrich UK (Merck Life Science UK Limited, Gillingham, Dorset, UK) and Abcam (Cambridge, UK), respectively. The active human HER-1 and HER-2 protein fragments, and the mouse anti-HER-2 monoclonal Ab were also purchased from Abcam (UK). The anti-goat antibody (Ab) conjugated to HRP, and the goat anti-HER-2 polyclonal Ab were purchased from R&D Systems (Abingdon, UK). The anti-rabbit IgG and anti-mouse IgG conjugated to HRP were purchased from Sigma Aldrich UK (Merck Life Science UK Limited, Gillingham, Dorset, UK). Incubation at 37 °C was performed using an HPLC oven model CT0-10AC Shimadzu.

Phosphate citrate buffer (0.05 M, pH 5.5) was prepared by dissolving one buffer tablet in 100 mL of distilled deionized water. TMB substrate solution was prepared by dissolving 1 mg of TMB in 150 µL of distilled water with hydrogen peroxide (1:10 dilution of 30% H_2_O_2_).

### 2.2. Fabrication of Screen-Printed Gold Electrode

The screen-printed gold electrodes (SPGE) used for the assay development were designed at Cranfield University and printed by DuPont Microcircuits Materials (Bristol, UK). The SPGE consisted of a silver/silver chloride (Ag/AgCl) reference electrode, a gold working electrode and a carbon counter. All three electrodes were fabricated using a procedure similar to that described by Noh and Tothill [44] and printed using the facilities at DuPont with inks provided by the company itself (DuPont Microcircuits Materials, Bristol, UK). The ink compositions used to produce the sensors were: carbon (BQ226), gold (BQ331), Ag/AgCl (5880) and encapsulation (5036 Blue) (DuPont Ltd., Bristol, UK). Electrodes were printed onto 125 µm thick polyethylene terephthalate (PET) sheets. While the base carbon print was dried by infrared light (IR light), the subsequent inks were box-oven dried at 140 °C for 30 min. The gold working electrode had a 5 mm diameter, giving a 19.6 mm^2^ planar area.

### 2.3. Electrochemical Measurements

The electrochemical measurements were carried out by dispensing a drop of 100 µL of a solution onto the SPGE, covering all three electrode areas (working, reference and counter). Each measurement was carried out in triplicates using a new electrode. For the electrochemical procedures, a computer-controlled four-channel Autolab analyser PGSTAT 10 from Autolab (Metrohm, UK) was used throughout the study. This allowed instantaneous reading of three-four sensors. The data were captured through the general-purpose electrochemical software GPES 4.9007 installed on the computer for single and multichannel readings. The SPGEs were connected to the Autolab using a PalmSens plugged boxed connectors purchased from Alvatek (Gloucestershire, UK). The boxed connectors were placed in a Faraday cage from Gamry Instruments (SciMed, Stockport, UK). Prior to each experiment, the sensors were cured at 120 °C for 30 min using a Carbolite oven (Hope, UK).

### 2.4. Optimization of the Capture and Detector HER-1 and HER-2 Antibodies on the Gold Electrode Surface

Solutions of capture mouse anti-HER-1 and anti-HER-2 monoclonal Abs (20 µL) at several concentrations (25–100 µg mL^−1^) were prepared separately in a carbonate-bicarbonate buffer (0.05 M, pH 9.6). The monoclonal Ab solutions (for HER-1 or HER-2) were dispensed on the gold working electrode surfaces individually and left for incubation overnight at 4 °C in a humid environment to achieve physical adsorption. After the incubation, the electrodes were washed with PBS containing 0.05% Tween 20 (PBS-T) followed by distilled water and dried with nitrogen gas. The electrodes were then blocked for 1 h at 37 °C using a blocking solution (100 µL of 1:10 milk concentrate in PBS), covering the entire SPGE surface area (working, reference and counter areas); they were then washed and dried at room temperature.

For the HER-1 sensor, an anti-species HRP was directly used for signal generation. For this, after washing and drying the blocked electrodes, 20 µL of anti-mouse-HRP (10 µg mL^−1^ prepared in PBS containing milk concentrate at 1:40 dilution) was added to the working electrode and incubated for 1 h at 37 °C. While for the HER-2 sensor, a solution of HER-2 antigen (20 µL of 150 ng mL^−1^, prepared in 1:40 dilution of milk concentrate in PBS) was dispensed on the working electrode and incubated for 1 h at 37 °C. After washing, 20 µL of goat anti-HER-2 polyclonal Ab (100 μg mL^−1^ prepared in PBS containing 1:40 milk concentrate) was dispensed on the working electrode surface and incubated for 1 h at 37 °C. Finally, after further washing, the anti-goat-Ab-HRP (10 μg mL^−1^, prepared in PBS containing 1:40 milk concentrate) was dropped on the electrode and incubated for 1 hr at 37 °C. For both the HER-1 and HER-2 assays, all electrochemical readings were performed by covering the entire SPGE surface with a solution of TMB (100 µL, 4 mM) and H_2_O_2_ (0.06%), prepared in 0.25 M citrate phosphate buffer, pH 5.5/0.5 M KCl. The chronoamperometry readings were then performed at −200 mV for 110 s.

The same protocol was used to optimize the detector antibody. In this case, fixed concentrations of 20 µL of HER-1 and HER-2 capture monoclonal mouse Ab (50 μg mL^−1^ for HER-1 and 75 μg mL^−1^ for HER-2), and of 20 µL of HER-1 and HER-2 antigen (800 ng mL^−1^ HER-1 and of 150 ng mL^−1^ HER-2) were used. Similarly, fixed concentrations of the anti-rabbit—HRP (20 μL of 10 μg mL^−1^ for HER-1) and anti-goat-HRP (20 μL of 10 μg mL^−1^ for HER-2) were also used, while varying the concentration of the detector rabbit polyclonal Ab (25–75 μg mL^−1^ for HER-1) and goat polyclonal Ab (50–100 μg mL^−1^ for HER-2).

To obtain a standard curve, various concentrations of HER-1 antigen (0.5–75 ng mL^−1^) and HER-2 antigen (5–200 ng mL^−1^) were tested. The procedure mentioned above was used with fixed concentrations of capture Ab (mouse monoclonal anti-HER-1 Ab, 20 μL of 50 μg mL^−1^ and mouse monoclonal anti- HER-2 Ab, 20 μL of 75 μg mL^−1^) and fixed concentrations of detector Ab, rabbit polyclonal antibody (20 μL of 50 μg mL^−1^ for HER-1), goat polyclonal Ab (20 μL of 75 μg mL^−1^ for HER-2). Finally, a fixed concentration of anti-rabbit-HRP (20 μL of 10 μg mL^−1^ for HER-1) and anti-goat-HRP (20 μL of 10 μg mL^−1^ for HER-2) was applied. The HER-1 and HER-2 assays were conducted independently from each other, and the results are reported separately. After obtaining the readings, the current was normalized by subtracting the control reading (reading in absence of antigen) and plotted vs. the HER-1 and HER-2 antigen concentrations. Linear regression and statistical calculations were performed using Excel and Graph Pad Prism. The limit of detection (LOD) and the limit of quantification (LOQ) were calculated as the antigen concentration equal to the average of the control readings (at least three readings) plus three times and ten times its standard deviation, respectively.

### 2.5. Assay Amplification Using Gold Nanoparticle

Colloidal gold nanoparticles (AuNPs, 40 nm) were employed here because of their efficacy in amplifying sensor signals, which can increase the sensitivity of assays and lower the limit of detection for the target analyte. The preparations of the AuNPs anti-rabbit Ab-HRP conjugate (for HER-1) and AuNPs anti-goat Ab-HRP conjugate (for HER-2) were conducted according to the procedure already described in the literature [43,44,45]. The AuNPs-Ab-HRP conjugate was prepared by adding 100 µL of anti-goat Ab—HRP (0.1 mg mL^−1^) and anti-rabbit Ab—HRP (1 mg mL^−1^) separately to a volume (1 mL) of AuNPs solution with a pH adjusted to pH 9 using 0.2 M sodium hydroxide (NaOH). These mixtures were then incubated at room temperature for 1 hr under gentle shaking. Once the incubation had finished, bovine serum albumin (BSA) solution (100 µL, 10% *w*/*v* in water) was added to the AuNPs-Ab-HRP mixtures and incubated for another hour at room temperature under gentle shaking. BSA was used to block the unoccupied surface of the AuNPs, and therefore, minimize the non-specific adsorption during the assay. The AuNPs-Ab-HRP conjugate obtained was then centrifuged at 10,000 rpm for 10 min at 4 °C. After centrifugation, the supernatants were discarded and the soft sediments of AuNPs-Ab-HRP conjugates were re-suspended in 70 µL of 0.01 M PBS and stored at 4 °C as a stock solution. The success of the conjugation reactions was tested by adding a drop of the AuNPs-Ab-HRP conjugate to a small volume (500 µL) of 2.5 M sodium chloride (NaCl). The solution turning purple, which is an indication of flocculation occurring, meant that the binding of the proteins to the gold particles had not been successful and the protocol needed to be repeated.

### 2.6. Dynamic Light Scattering (DLS) Size Analysis before and after Gold Nanoparticle Conjugation

The measurement of AuNPs before and after conjugation with Ab was carried out using Dynamic Light Scattering (DLS) (Malvern Zetasizer, Malvern Instruments Ltd., Malvern, UK). The measurements were carried out in 3 cm^3^ disposable polystyrene cuvettes with a sample volume of 1 mL at 25 °C, according to the manufacturer’s instructions. Each sample was measured in triplicate and the mean value was reported.

### 2.7. Transmission Electron Microscopy (TEM) before and after Gold Nanoparticle Conjugation

Transmission Electron Microscopy (TEM) images of the AuNPs before and after conjugation with Ab were taken using a Philips CM 20 Transmission Electron Microscope. Prior to the imaging, the samples were dispensed onto silicon chips attached to the TEM holder and left to dry overnight in a fume hood.

### 2.8. Detection of HER-1 and HER-2 Using the AuNP-Ab-HRP Conjugate in the Indirect Sandwich Assay

A dilution of the AuNPs-Ab-HRP conjugates (10 times dilution in PBS, pH 7.4, 0.01 M), prepared as explained in Section 2.5, was used in the indirect ELISA sandwich assay. The optimized assay was tested against various concentrations of HER-1 antigen (0–0.5 ng mL^−1^) and HER-2 antigen (0–50 ng mL^−1^). The indirect ELISA format was performed as described in Section 2.4 with capture antibody concentrations of 50 µg mL^−1^ (HER-1) and of 75 µg mL^−1^ (HER-2), followed by the antigens (HER-1 or HER-2) then by 50 µg mL^−1^ (HER-1) and 75 µg mL^−1^ (HER-2) polyclonal antibody and finally by 1:10 dilution of AuNP-Ab-HRP for signal generation (instead of the anti-goat and anti-rabbit Ab-HRP conjugate). After the chronoamperometry readings at −200 mV, the current was normalized by subtracting the control reading (absence of antigen) and plotted vs. the HER-1 and HER-2 antigen concentration in two separate datasets. Linear regression and statistical calculations were performed. The LODs and LOQs were calculated as the concentration equal to the average of the control readings plus three and ten times its standard deviation, respectively. The HER-1 and HER-2 assays were conducted independently from each other.

### 2.9. Optimization of the Commercial Serum for the Indirect Assay

To study the impact of using serum instead of buffer solutions for the assay, commercial human serum from Sigma Aldrich UK, (Merck Life Science UK Limited, Gillingham, Dorset, UK) at different concentrations (50–75%) was prepared in PBS (pH 7.4, 0.01 M). Samples of 100% human serum (without the addition of buffer) were also investigated.

### 2.10. Detection of HER-1 and HER-2 in 100% Commercial Serum Using the Standard Assay Format

**Scheme 1:**
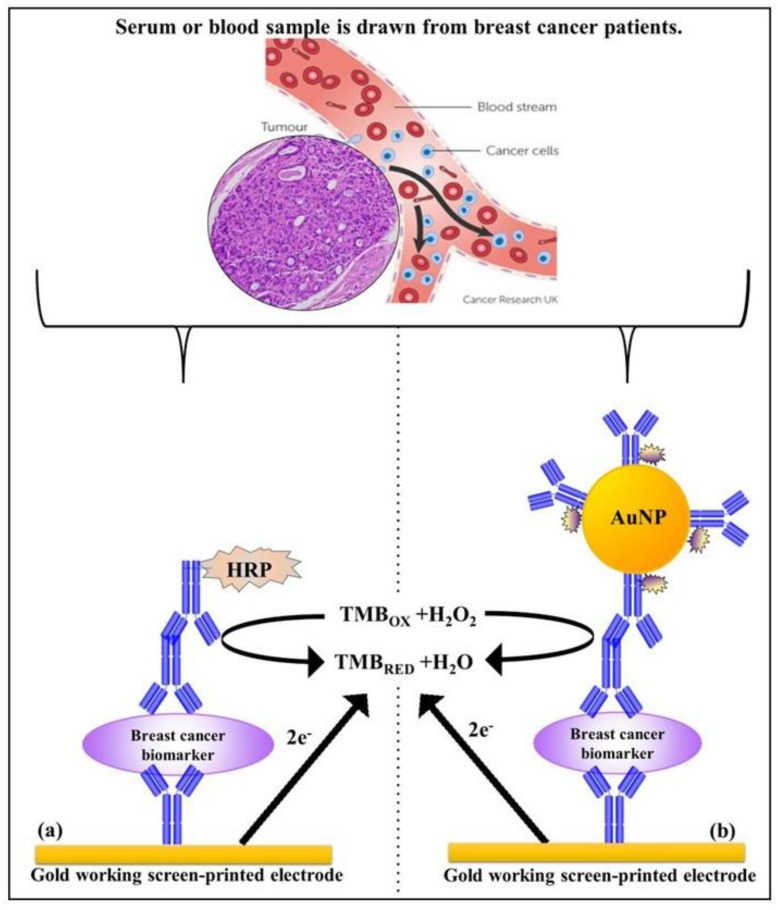
Principle of the indirect sandwich assays for the biomarker detection on the gold Sensor chip; (**a**) standard assay and (**b**) gold nanoparticle assay.

The performance of the standard indirect assay using anti-rabbit (HER-1) and anti-goat (HER-2) Ab-HRP with samples spiked in 100% serum was tested. The assay was performed by spiking undiluted (100%) human serum with concentrations of HER-1 in the range of 0–75 ng mL^−1^ and HER-2 in the range of 0–80 ng mL^−1^. The indirect ELISA format was then performed as described in Section 2.4 with fixed concentrations of the capture anti-HER-1 Ab (50 µg mL^−1^) and capture anti-HER-2 Ab (75 µg mL^−1^). This was followed by the spiked serum samples, and then by the detector Ab consisting of polyclonal anti-HER-1-Ab (50 µg mL^−1^), and polyclonal anti-HER-2 Ab (75 µg mL^−1^), and finally by the anti-rabbit (HER-1) and anti-goat (HER-2) Ab-HRP conjugate (10 µg mL^−1^) (standard assay) for signal generation. For HER-2, experiments with spiked serum samples were also performed using 1:10 dilution of the AuNPs-Ab-HRP conjugate rather than the anti-goat-Ab-HRP. As before, the LODs and LOQs were calculated as the concentration equal to the average control readings plus three and ten times its standard deviation, respectively. Schemes for the indirect and the indirect AuNPs enhanced sandwich sensor for HER-1 and HER-2 are shown in Scheme 1.

### 2.11. Cross-Reactivity Studies for HER-2 Sensor

The specificity of the HER-2 immunosensor was investigated by testing the HER-1 antigen, using the indirect AuNPs enhanced assay. Following the procedure described in Section 2.4, a fixed concentration of capture anti-HER-2 Ab (75 µg mL^−1^) was immobilized on the gold surface and the sensor was then incubated for 1 h at 37 °C with a fixed concentration of the sample, HER-1 (75 ng mL^−1^). The HER-1 sample was prepared and tested both in buffer and undiluted serum (100% serum). A fixed concentration of detector polyclonal Ab (75 µg mL^−1^) and finally, a 1:10 dilution of AuNPs-Ab-HRP conjugate were used for signal generation.

## 3. Results

### 3.1. Characterization and Optimization of the Sensor Signal

The electrochemical immunosensors developed in this work were based on an indirect sandwich ELISA format applied on screen-printed gold electrodes (SPGE) with HRP as the enzyme label and TMB/H_2_O_2_ as the substrate/mediator system. Cyclic voltammetry and chronoamperometry were used to characterize the electrodes and to optimize the electrochemical conditions for the measurements, as already explained in our previous work [42]. The characterization of the SPGE evidenced an active surface area of the gold working electrode (Active %) equal to 81%, while the best ratio signals current to background current, achieved using step-amperometry [46], was obtained while applying a potential of −200 mV. Therefore, this potential was selected for future immunosensor development and for further amperometric measurements.

### 3.2. Development of the HER-1 and HER-2 Immunosensor

The assay format used throughout the development of the immunosensors was an indirect sandwich ELISA (Scheme 1) with or without gold nanoparticles. Two monoclonal and two polyclonal antibodies against HER-1 and HER-2 were used as the capture and detector antibody, respectively. Anti-rabbit/mouse antibodies raised in goat conjugated to HRP were used for HER-1 and an anti-goat Ab raised in donkey conjugated to HRP was used for HER-2, for signal generation.

Each antibody concentration was optimized independently to obtain the best sensor performance. Therefore, different concentrations of capture mouse monoclonal anti-HER-1 and anti-HER-2 Ab (0, 25, 75, 50 and 100 μg mL^−1^) were tested on the sensor surface independently via overnight adsorption. For HER–2 a full assay format was employed for the optimization, by fixing the concentration of the HER-2 antigen at 150 ng mL^−1^ and keeping constant the concentration of the detector polyclonal Ab at 100 µg mL^−1^. For signal generation, the anti-goat Ab-HRP conjugate (anti-species) was also kept at a fixed value of 10 µg mL^−1^. However, for HER-1, instead of using the whole assay, and to reduce the costs, following immobilization of the capture Ab and the sensor surface blocking, an anti-species conjugated with HRP was directly used for signal generation. The anti-species antibody used was an anti-mouse antibody (Sigma Aldrich UK, Merck Life Science UK Limited, Gillingham, Dorset, UK), raised in goat conjugated to HRP at a concentration of 10 µg mL^−1^.

**Figure 1:**
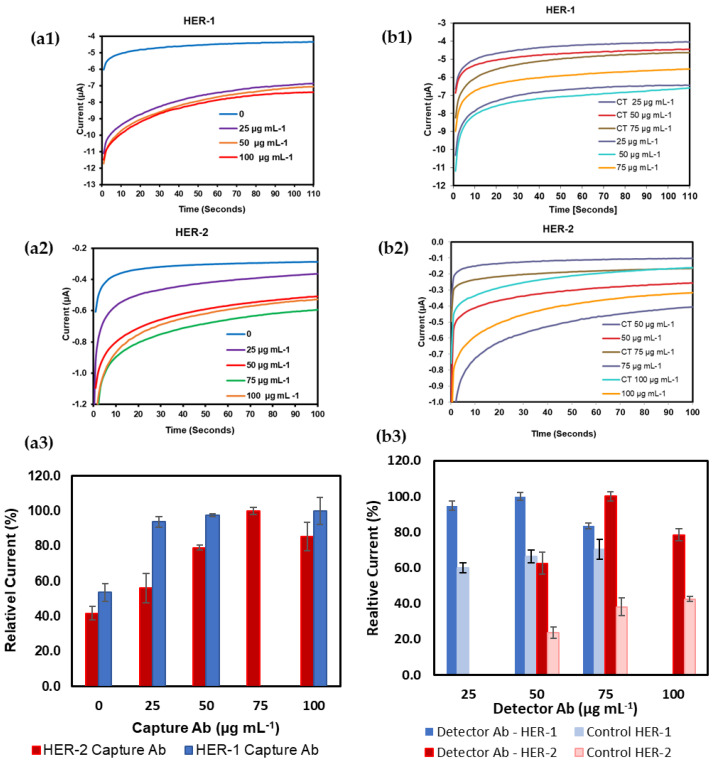
Chronoamperometric signal for SPGE; (**a1**) different concentrations of monoclonal anti-HER-1; (**a2**) anti-HER-2 as the capture antibody; (**a3**) the relative current percentage calculated for each concentration for both HER-1 and HER-2; (**b1**) different concentrations of polyclonal anti-HER-1; (**b2**) anti-HER-2 as the detector antibody; (**b3**) the relative current percentage calculated for each concentration for both HER-1 and HER-2. Standard deviations were calculated for experiments performed in triplicate. In (**b1**,**b2**) CT stands for control readings.

Figure 1(a1,a2) shows the original changes in current as the concentration of the capture Ab for both HER-1 and HER-2 was increased. Further to this, the relative current percentage was calculated for each concentration for both HER-1 and HER-2 to see the difference in the activity (Figure 1(a3)). These results show that saturation was reached at a capture Ab concentration of 50 µg mL^−1^ for HER-1 and 75 µg mL^−1^ for HER-2. Additionally, no further increase in signal beyond these concentrations was observed. However, particularly for HER-2, a reduction in signal was shown when higher concentrations of capture Ab were tested (100 µg mL^−1^). This can be attributed to steric hindrance [47], which can result in a layer of capture Ab being too tightly packed on the sensor surface, hindering the binding of the antigen to the antibody active sites. Therefore, from the results observed, it was concluded that a concentration of capture Ab of 50 µg mL^−1^ for HER-1 and 75 µg mL^−1^ for HER-2 produced the best response and these were selected for future assay development. Subsequently, in order to optimize the concentration of the detector Ab, after immobilizing the optimized concentrations of capture Abs for both HER-1 and HER-2 and by keeping constant the concentration of the antigens (HER-1 at 800 ng mL^−1^ and HER-2 at 150 ng mL^−1^) the concentration of the detector Ab was varied between 25 and 75 µg mL^−1^ for HER-1 and 50 and 100 µg mL^−1^ for HER-2 (Figure 1b). Figure 1(b1,b2), shows the current signals recorded for HER-1 and HER-2, respectively. The current recorded in absence of the antigen ‘Control’ is also shown and this was used to assess the concentration of detector Ab that was able to generate not only the highest signals, but also the most specific sensor response (greatest difference between presence and absence of antigens). Further to this, Figure 1(b3), shows the same current signals but calculated as a relative current percentage for a more direct comparison.

From these results, it can be observed that the concentration of HER-1 polyclonal Ab of 50 μg mL^−1^ produced a slightly higher relative current percentage than 25 μg mL^−1^. However, the highest concentration of polyclonal Ab tested (75 μg mL^−1^) was seen to show a drop in the relative current percentage, with a lower difference compared to the control signal. Therefore, from the results, it was decided to use 50 µg mL^−1^ for the HER-1 detector Ab and this was selected for future assay work. Similarly, for HER-2, the results showed that the concentration of polyclonal Ab of 75 μg mL^−1^ produced the best signal (highest current and greatest difference compared to the control relative current percentage). Again, the highest concentration of polyclonal Ab tested (100 μg mL^−1^) was seen to produce a drop in sensor signal compared to the lower one (50 μg mL^−1^) with a lower signal difference compared to the control. Again, the reason for the worse results obtained with the highest concentration of Ab tested can be attributed to steric hindrance [47]. Therefore, from these results, for HER-2, 75 µg mL^−1^ was chosen as the best concentration for the detector Ab and selected for future assay work.

The blocking of the sensor was also optimized to ensure low non-specific binding on the sensor surface after attaching the capture antibody and applying the full immunoassay. Solutions of 1% BSA, 1% Gelatin and 1:1, 1:5, 1:10 and 1:20 milk diluent in PBS pH 7.4 were tested. The best results were achieved when 1:10 milk diluent in PBS pH 7.4 was used.

### 3.3. Standard Plot for the Optimized HER-1 and HER-2 Immunosensor in Buffer Samples

**Figure 2:**
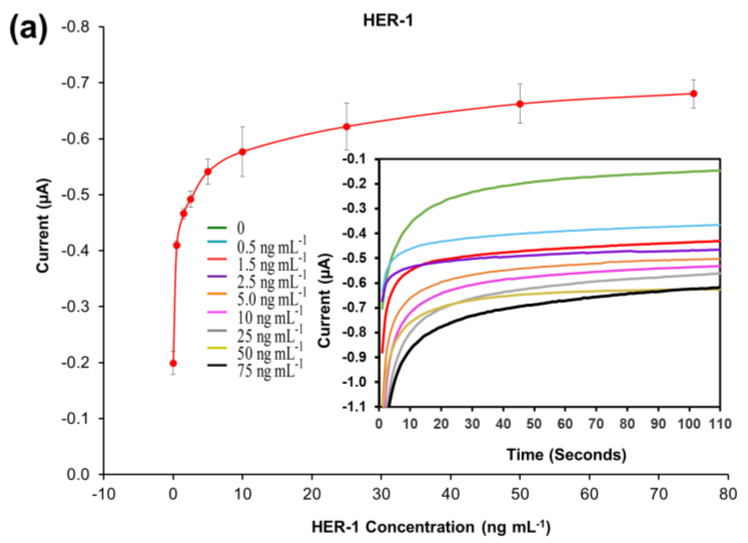

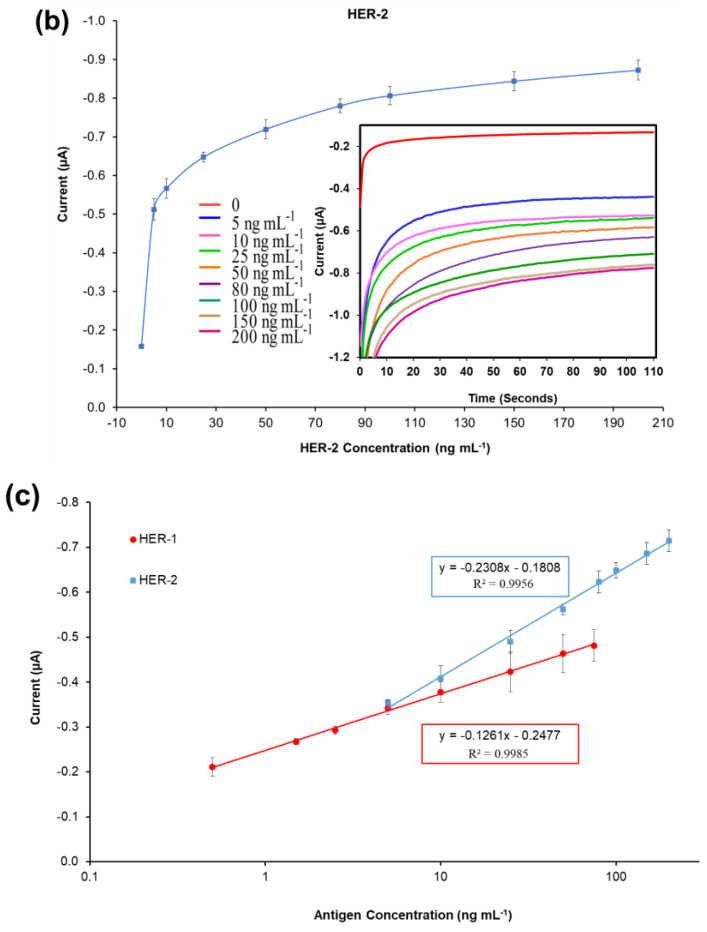
Biosensors responses of the optimized immunoassays against various concentrations of; (**a**) HER-1 antigen (0.5–75 ng mL^−1^); (**b**) HER-2 antigen (5–200 ng mL^−1^). Measurements were taken after 110 s at a potential of −200 mV. Embedded graphs show the raw data obtained. (**c**) Linear regression of the HER-1 and HER-2 assay, obtained with data taken from Figure 2a,b. Standard deviations were calculated for experiments performed in triplicate (*n* = 3).

A standard plot for the HER-1 and HER-2 immunosensors was constructed based on the indirect ELISA format by testing the biosensor response to HER-1 and HER-2 independently. Concentrations ranging from 0.5 to 75 ng mL^−1^ (HER-1) and 5 to 200 ng mL^−1^ (HER-2) prepared in PBS buffer were tested (Figure 2a). The assay was performed by keeping constant the concentrations of the capture Ab (50 μg mL^−1^ for HER-1 and 75 μg mL^−1^ for HER-2), followed by the detector Ab (50 μg mL^−1^ for HER-1 and 75 μg mL^−1^ for HER-2), and finally the anti-species—HRP Ab (10 µg mL^−1^). For the control, readings were carried out in the absence of the antigen, which was replaced by milk: PBS buffer diluent (1:40). It can be observed in Figure 2a,b, that when HER-1 and HER-2 antigen concentration increased, the resulting sensor signal also augmented (μA), indicating that the increase in current is proportional to the HER-1 and HER-2 concentration. The current values recorded were then normalized by subtracting the control readings and plotted against the antigen’s concentration in a linear regression graph with a log x-axis (Figure 2c). Figure 2c shows that the sensors’ responses are linear in the concentration ranges tested. Based on the equation formula, the calculated limit of detection (LOD, control readings +3× SD) and the limit of quantification (LOD, control readings +10× SD) for HER-1 were as low as 1.06 ng mL^−1^ and 2.1 ng mL^−1^ (R^2^ of 0.998) in buffer samples. This LOD is more sensitive and lower than that of two commercial HER-1 ELISA kits, which, when tested, showed a LOD of 300 ng mL^−1^ (human HER-1 full-length ELISA kit, Invitrogen, Thermo Fisher UK, Loughborough, UK) and 1.25 ng mL^−1^ (Human HER-1 Sandwich ELISA Kit, Life Span Biosciences Inc., Seattle, USA). It has been reported by Li and co-workers [11] that the biomarker level in healthy individuals is in the range of about 1.0–25 ng mL^−1^, while between the primary diagnostic step and metastatic diagnostic step, it rises to around 11–30 ng mL^−1^ and is up to 270 ng mL^−1^ in women with breast cancer. Therefore, the LOD of this newly developed sensor falls within the clinical range demonstrating a potential for medical applications.

Similarly, for HER-2, based on the calibration curve reported in Figure 2c, the LOD and the LOQ were calculated to be 0.95 ng mL^−1^ and 1.5 ng mL^−1^ (R^2^ of 0.996). Compared to HER-1 results the LOD achieved here for HER-2 is slightly higher than those of commercial HER-2 ELISA kits, which were around 0.312 ng mL^−1^ for the HER-2 sandwich ELISA kit (Life Span Biosciences Inc., Seattle, USA) and 0.2 ng mL^−1^ for HER-2 total ELISA kit (Thermo Fischer UK, Loughborough, UK). Even though the sensitivity of this newly introduced sensor is not as low as the commercially available ELISA kits, it can be argued that the HER-2 biomarker does not require such low LODs, as its physiological range is between 2.0 and 15 ng mL^−1^ for healthy individuals, while it rises to a range between 15 and 75 ng mL^−1^ in women with breast cancer [48,49].

Therefore, the working range of both electrochemical immunosensors developed here precisely covers the critical concentration ranges for the biomarkers, showing a potential to be used as a future method for quantification of HER-1 (EGFR) and HER-2, and for the possible detection of breast cancer in patients. Nevertheless, if for any reason a need for more sensitive immunosensors arises, an enhancement of the sensor signal for the biomarkers can be easily achieved using gold nanoparticles, as these could increase the sensitivity and lower the sensors working ranges and limits of detection.

### 3.4. Evaluation of Gold Nanoparticles as a Technique for Signal Enhancement Using Buffer Samples

Interest in the exploitation of gold nanoparticles (AuNPs) in this work was due to their capability to increase specific surface areas and to their easy bioconjugation that does not require chemical modifications [40,41]. Furthermore, AuNPs can amplify sensor signals due to their ability to adsorb more than one labelling molecule on their surface [40,42]. Thus, the signal created per bound antibody is amplified compared to the conventional indirect format (Scheme 1).

**Figure 3:**
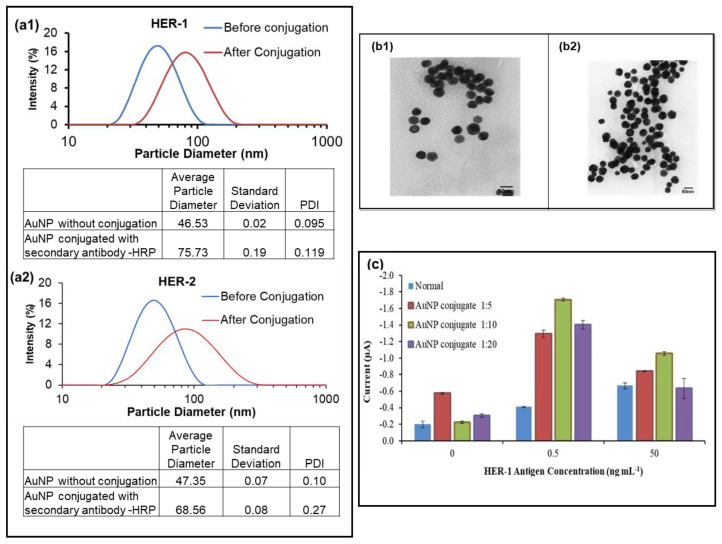
The DLS results of the AuNP, before and after conjugation; (**a1**), HER-1; (**a2**), HER-2. Standard deviations were calculated for experiments performed in triplicate. (**b1**), TEM imaging results of the gold nanoparticle before conjugation; (**b2**), after conjugation with anti-rabbit -HRP; (**c**) HER-1, used here as an example of data achieved using different AuNP conjugate dilution (*n* = 3).

Hence, the use of AuNPs for the sensor assay was investigated to enhance the signal and amplify the sensitivity of both immunosensors. The anti-species-HRP Ab (secondary antibody) was replaced with AuNP conjugated with the anti-rabbit-HRP Ab (for HER-1) and anti-goat-HRP Ab (for HER-2). The adsorption of the anti-goat and rabbit Ab-HRP onto the AuNPs was aided by a combination of ionic and hydrophobic interactions between the Ab-HRP molecules (negatively charged) and AuNPs (positively charged). To observe that the conjugation was successful, dynamic light scattering (DLS) was conducted on the AuNP before and after conjugation with the anti-species Ab. Figure 3(a1,a2) shows that the HER-1 and HER-2 conjugation was considered successful, as a shift was seen in the size of the AuNP from 46.53 ± 0.02 nm before to 75.73 ± 0.19 nm after conjugation for HER-1 and from 47.35 ± 0.07 nm before to 68.56 ± 0.08 nm after conjugation for HER-2. The shift observed was due to the adsorption of the anti-species Ab labelled with HRP onto the gold nanoparticle, increasing the diameter of the AuNP. Figure 3(b1,b2) shows the TEM images of the AuNP before and after conjugation with the anti-rabbit-HRP Ab (for HER-1).

Once the conjugation method was established, the AuNP-Ab-HRP conjugates were tested using various dilution factors (1:5, 1:10 and 1:20) in PBS to find the best concentration to use for the sensor. While the AuNP-Ab-HRP conjugate concentration and the antigen concentration varied, all the other parameters were kept the same. The concentrations of the antigens (0, 0.5 and 50 ng ml^−1^) were selected based on the results obtained from the buffer samples assay. Figure 3c, which depicts the results obtained for HER-1, shows that the use of AuNP proved to enhance the signal compared to the ‘normal’ assay. In addition, although all three dilutions produced higher readings than the normal assay, the control reading gave the highest value when the most concentrated dilution of the AuNP conjugate was tested (1:5 dilutions). Furthermore, it could be clearly seen that the 1:10 dilution factor generated the best results in terms of the control and the amplification. Another significant finding shown in Figure 3c was the fact that the AuNP-Ab-HRP conjugate assay reacted differently than the normal assay, giving a higher signal at 0.5 ng mL^−1^ for all three-dilution factors compared to 50 ng mL^−1^. This indicates that the AuNP-conjugate has not only amplified the signal, but has also made the assay more sensitive and, at the highest antigen concentration, the assay may have already reached saturation. Additionally, steric hindrance may be involved in limiting further binding events. Therefore, the 1:10 dilution factor was selected for future AuNP-Ab-HRP-based assays for both HER-1 and HER-2.

### 3.5. Standard Plot for the Optimized AuNP-Ab-HRP Conjugate Immunosensor Using Buffer Samples

**Figure 4:**
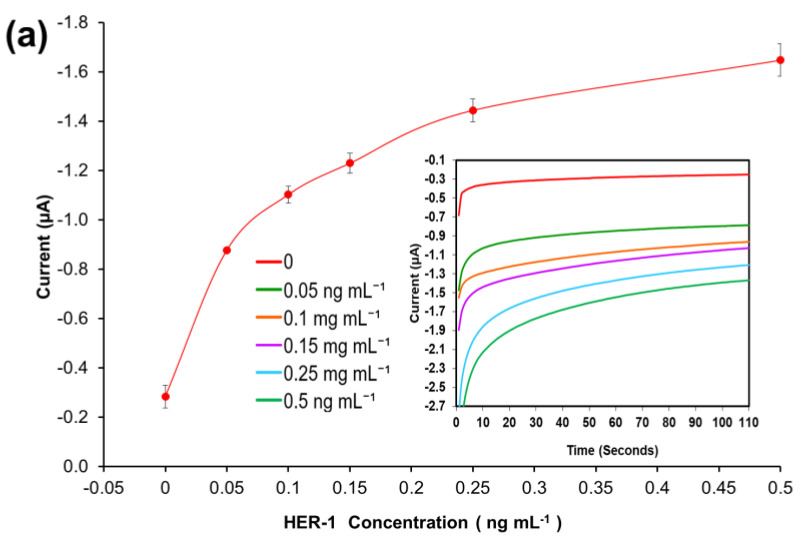

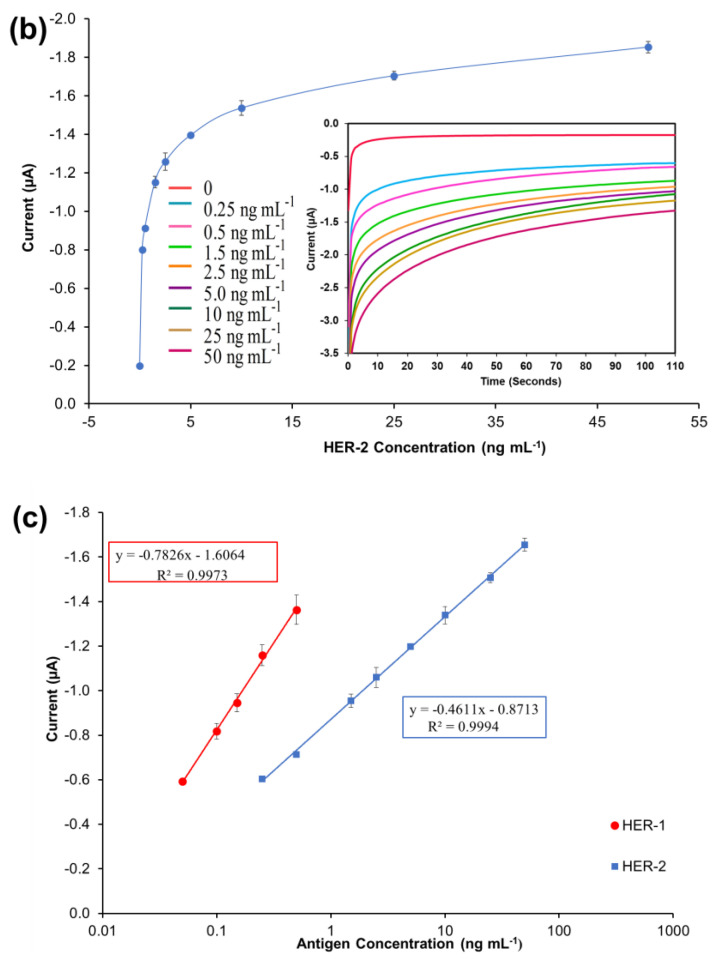
Biosensor response of the optimized immunoassay performed using AuNP-Ab-HRP conjugates against various concentrations of; (**a**) HER-1 antigen (0–0.5 ng mL^−1^) and (**b**) HER-2 antigen (0–50 ng mL^−1^). The embedded graphs show the raw data reading obtained from the chronoamperometry measurements. (**c**) Linear regression curves for HER-1 and HER-2 AuNP-Ab-HRP conjugate assay, obtained with data taken from Figure 4a,b. Standard deviations were calculated for experiments performed in triplicates (*n* = 3).

A standard plot for the HER-1 and HER-2 immunosensor using 1:10 AuNPs-Ab-HRP conjugates was constructed based on the indirect ELISA format by testing the biosensor’s response to HER-1 and HER-2 independently. Concentrations ranging from 0.05 to 0.5 ng mL^−1^ for HER-1 (Figure 4a) and from 0.25 to 50 ng mL^—1^ for HER-2 (Figure 4b) were used. The assays were performed while keeping constant the concentrations of the capture Ab (50 μg mL^−1^ for HER-1 and 75 μg mL^−1^ for HER-2) followed by the detector Ab (50 μg mL^−1^ for HER-1 and 75 μg mL^−1^ for HER-2) and finally by the AuNP-Ab-HRP conjugates (1:10 dilution factor).

Figure 4a,b, shows that using the AuNP has improved the HER-1 and HER-2 assay sensitivity as well as amplifying the signal as, in general, higher current values were recorded. Unlike the normal buffer assay, it was found for HER-1 that the AuNP-Ab-HRP-based assay was saturated by 0.5 ng mL^−1^ (Figure 4a). Furthermore, unlike the AuNP-Ab-HRP conjugate assay, the normal buffer assay showed a gradual increase in the signal beyond 0.5 ng mL^−1^ and the immunosensor could detect the antigen up to 75 ng mL^−1^. Similarly, Figure 4b shows that the use of the AuNPs-Ab-HRP conjugate improved the HER-2 assay sensitivity and amplified the sensor signal as compared with the assay performed without AuNP. Unlike the ‘normal’ buffer assay, which showed an increase in the signal between 5 ng mL^−1^ and 200 ng mL^−1^ of antigen, the assay conducted using the AuNP-Ab-HRP conjugate showed an increase in signal between 0.25 ng mL^−1^ and 50 ng mL^−1^. However, beyond this concentration (50 ng mL^−1^) the assay signal started decreasing (results not shown), probably due to steric hindrance [47]. Therefore, for both antigens, the use of the AuNPs-Ab-HRP conjugate has been shown to amplify the sensor signal and increased the sensitivity of the assay compared to the normal buffer assay. The reason behind the increased signal is most likely due to a higher number of HRP molecules being available for signal generation, as multiple copies of the anti-species Ab-HRP conjugate are attached to each individual AuNP.

Figure 4c depicts the current normalized by subtracting the control reading and plotting against the HER-1 and HER-2 concentration in a linear regression graph with a log x-axis. Based on the equation formula in Figure 4c, the LODs calculated as explained previously were found to be, for both immunosensors, as low as 30 pg mL^−1^, whereas the LOQs were 98 pg mL^−1^ and 35 pg mL^−1^ for HER-1 and HER-2, respectively (R^2^ = 0.997 for HER-1 and R^2^ = 0.999 for HER-2). For the HER-1 immunosensor, the clinical samples might need to be diluted to match the linear range of the AuNP-conjugate sensor (HER-1 serum concentration range for healthy patients is between 1.0 and 25 ng mL^−1^ and it is higher in breast cancer patients). Conversely, for HER-2, the AuNP-conjugate sensor is suitable to detect the biomarkers as the immunosensor covers the critical and lower serum concentration (2.0–15 ng mL^−1^) for healthy patients, and most of the concentration range in women with breast cancer (15–75 ng mL^−1^) [48]. Therefore, these new improved sensors show potential to be used as a future detection method for breast cancer patients, due to their ease of use. However, to have a full assessment of the sensor performance, tests needed to be carried out in biological fluids to evaluate both the sensitivity and whether the use of serum would interfere with the assay. Therefore, the analysis of commercial serum samples spiked with HER-1 and HER-2 was conducted.

### 3.6. Detection of HER-1 and HER-2 in Serum Samples Using ‘Normal’ Assay

**Figure 5:**
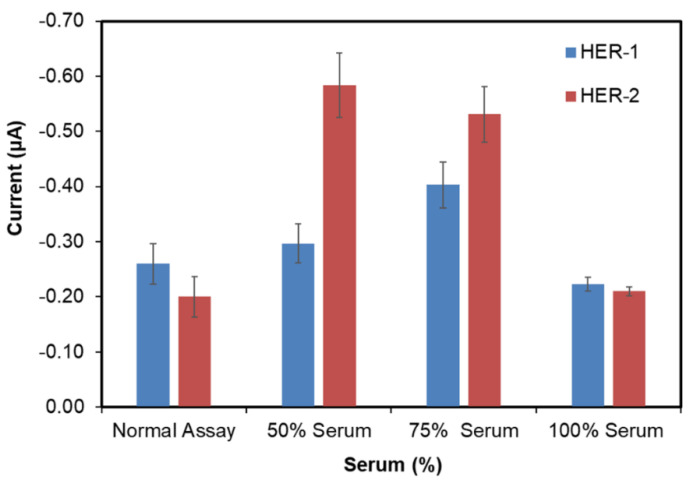
Serum samples concentrations (50, 75 and 100%) with no HER-1 and HER-2 antigen applied to the immunosensor assay. Standard deviation is for experiments performed in triplicates (*n* = 3).

In order to develop a clinically relevant sensor, it is essential to validate the assay in the biological fluid of choice (i.e., human serum). Therefore, in this work commercial serum both diluted (50% and 75%) and undiluted (100%) was tested, where to obtain 50% and 75%, the serum was diluted with PBS. All three serum dilutions were first tested with no antigen to evaluate the non-specific binding. From Figure 5, it can be observed that the 100% serum showed the lowest signal for both HER-1(−0.22 μA) and HER-2 (−0.20 μA), compared to 50% (−0.3 μA for HER-1 and −0.6 μA for HER-2) and 75% (−0.4 μA for HER-1 and −0.5 μA for HER-2) serum. Furthermore, when comparing the signal produced by 100% serum and the signal produced from the normal (buffer) assay control (−0.26 μA for HER-1 and −0.16 μA for HER-2), the interference of the serum seemed to be low as both results appear very similar. The reason behind the low interference of whole serum (100% serum) in the electrochemical immunosensors might be due to the ability of some of the components in the serum to further block the surface of the sensor, minimizing the interference from the serum itself. Such components are not as concentrated, and therefore, not as effective when serum is diluted with PBS. This shows the advantage of using screen-printed electrodes and chronoamperometry when compared to non-labelled techniques such as Surface Plasmon Resonance (SPR) and Quartz crystal microbalance (QCM), which require much better control of non-specific binding [43,50]. Hence as the result obtained for undiluted serum showed the lowest non-specific adsorption, it was decided that this would be used for future serum assay work for both the normal and the gold nanoparticle serum assay.

**Figure 6:**
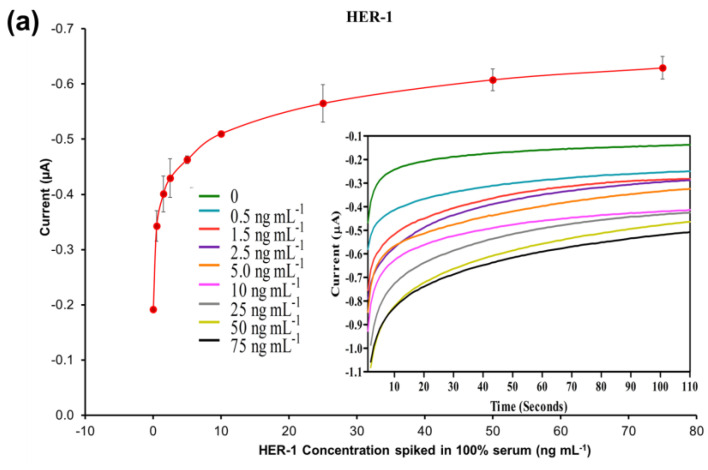

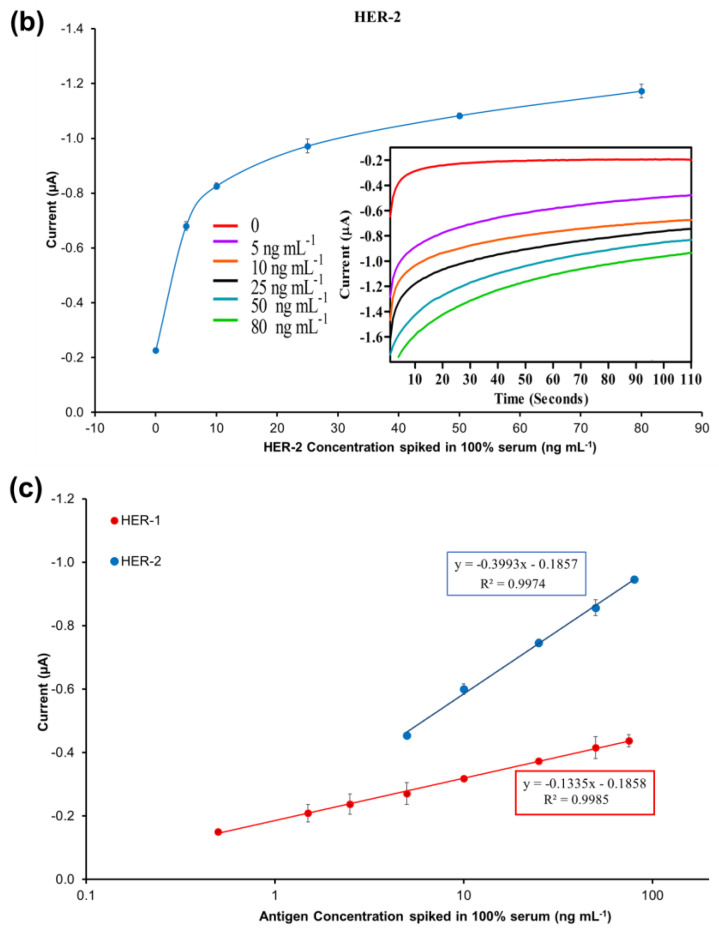
Standardized biosensor response of the optimized immunoassay against increasing concentrations of (**a**) HER-1 antigen (0.5–75 ng mL ^−1^); (**b**) HER-2 antigen (5–80 ng mL^−1^) spiked in 100% serum. Embedded graph shows the raw chronoamperometry measurements. (**c**) Linear regression of the assay spiked in serum samples with data taken from Figure 6a,b. Standard deviations were calculated from experiments performed in triplicate (*n* = 3).

To evaluate the feasibility of the developed electrochemical immunosensor for use with serum samples, the HER-1 and HER-2 indirect ELISA sandwich immunoassays were conducted independently using the previously optimized conditions and within the range of 0.5–75 ng mL^−1^ for HER-1 and 5–80 ng mL^−1^ for HER-2 spiked in 100% serum. Figure 6a,b show that the control readings (absence of biomarkers) in 100% human serum were −0.19 μA for HER-1 and −0.22 μA for HER-2, which are low and similar to the control readings of the buffer assay, evidencing a low level of non-specific binding. Thus, the linear regression curves plotted with a log-x axis, in Figure 6c, were obtained after the subtraction of the non-specific binding response.

Based on the equation formula in Figure 6c, for HER-1 a LOD of 1.2 ng mL^−1^ and a LOQ of 1.5 ng mL^−1^ (R^2^ of 0.999) were calculated. The LOD in the serum samples is slightly higher than the buffer samples (1.06 ng mL^−1^). Nevertheless, even though the sensitivity decreased for the serum assay, the electrochemical immunosensor is still suitable to detect HER-1 biomarkers in breast cancer patients, as it covered the full critical clinical concentration range (i.e., 1.0–25 ng mL^−1^ in healthy people and 11–30 ngmL^−1^ in patients with primary and metastatic cancer). Therefore, the sensor can productively distinguish between healthy and cancerous patient samples.

For HER-2, the LOD and the LOQ were found to be 1.47 ng mL^−1^ and 2.1 ng mL^−1^, respectively (R^2^ of 0.997). Similar to HER-1, for HER-2 there was a slight deterioration of both LOD and LOQ (0.52–0.60 ng mL^−1^ higher) as compared to the assay performed with buffer samples. The small loss in performance may be due to the worse signal-to-noise ratio resulting from a higher amount of non-specific binding of the serum on the sensor assay. For HER-2, the detection range was also reduced from 5–200 to 5–80 ng mL^−1^, as the assay showed saturation at 80 ng mL^−1^ (beyond this the assay signal decreased; results not shown). The reason behind the earlier saturation of the assay in serum may be due to a partial blockage of some of the capture Ab recognition sites on the electrode surface, hence reducing the binding of the antigen. Nevertheless, this new detection range does not affect the use of the immunosensor for clinical applications as it still covers the full range from healthy to cancer-suffering patients (2–75 ng mL^−1^).

In the future, further tests can be conducted with random patient serum samples and healthy samples for proof of concept and to further validate the two immunosensors. In addition, a microfluidic input could be applied to the electrochemical immunosensor to produce full-functioning biosensors for HER-1 and HER-2 detection independently and in combination in breast cancer.

### 3.7. Evaluation of the AuNP Amplified Immunosensor in Serum Samples and Cross-Reactivity Studies

The HER-2 immunosensor using the AuNPs-Ab-HRP conjugate instead of the anti-goat Ab-HRP conjugate for a signal generation was also tested in whole serum (100%) to evaluate its feasibility for clinical applications. Therefore, the indirect sandwich immunoassay was carried out with the conditions optimized previously testing 100% serum spiked with 0.25–25 ng mL^−1^ of the HER-2 biomarker. The test was only performed for HER-2, as when performed in buffer, the HER-1 AuNP-Ab-HRP conjugate assay was already too sensitive and out of the detection range. Therefore, it was decided that the amplification of the assay with the AuNP was not needed for the quantification of HER-1 in serum samples, reducing costs and saving time.

**Figure 7:**
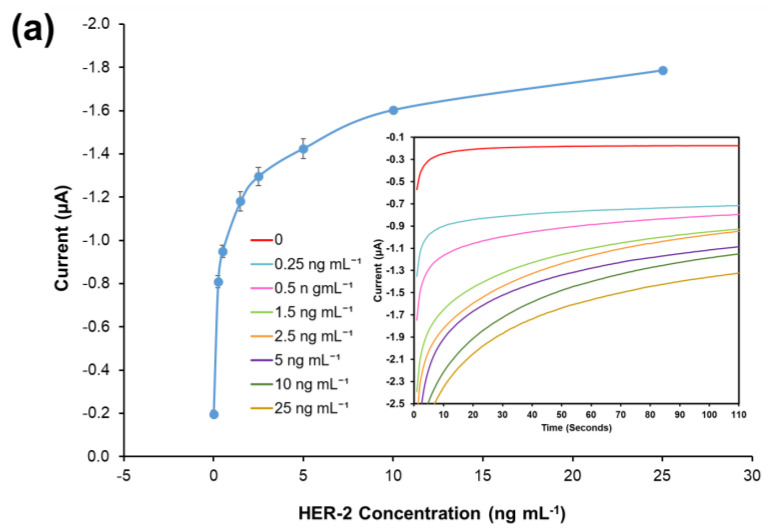

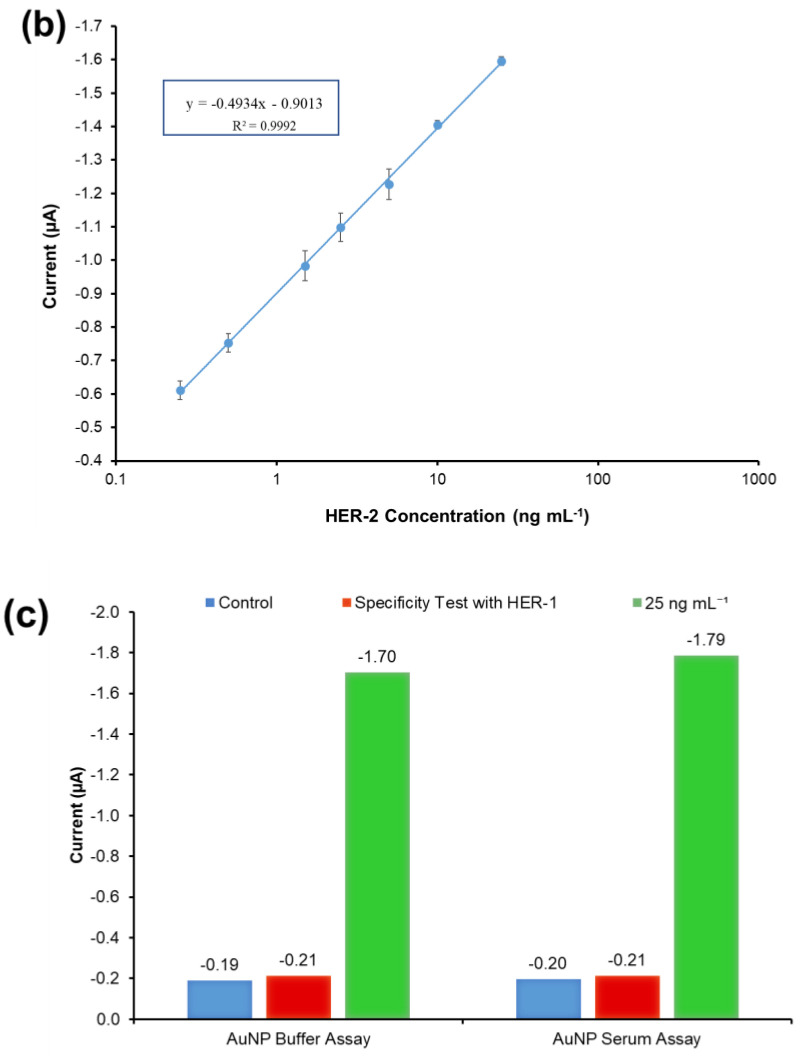
(**a**) Standardized biosensor response of the optimized immunoassay against various concentrations of HER-2 antigen spiked in 100% serum (5–25 ng mL^−1^); the embedded graph shows the raw chronoamperometry measurements. (**b**) Linear regression of the AuNP-Ab-HRP conjugate assay spiked in serum samples obtained using data taken from Figure 7a. (**c**) Cross-reactivity study of the HER-2 sensor for HER-1 *via* the indirect ELISA sandwich assay performed using AuNPs-Ab-HRP conjugate as a signal amplification in both buffer and 100% serum. Standard deviations were calculated for experiments performed in triplicate (*n* = 3).

The results illustrated in Figure 7a show that the non-specific binding of whole serum (without spiked HER-2) was low (−0.197 μA), and very similar to the value achieved when the assay was performed with buffer (−0.190 μA). The figure also shows that as usual an increment in sensor signal proportional to the concentration of the biomarker was observed. The linear regression graph obtained by plotting with a log-x axis and after subtraction of the response of the blank (control) undiluted serum is reported in Figure 7b. Based on the equation formula in Figure 7b, the LOD and the LOQ were calculated as 50 pg mL^−1^ and 80 pg mL^−1^, respectively (R^2^ of 0.999). The detection limit is seen to be only 20 pg mL^−1^ higher than the AuNP immunosensor performed in the buffer, showing a minimal effect from the sample matrix.

Moreover, the sensor detection range in serum was reduced to 0.25–25 ng mL^−1^ from the range of 0.25–50 ng mL^−1^ observed in the buffer. In fact, the sensor signal saturation in serum was reached by 25 ng mL^−1^ of HER-2 as no further increase was recorded for higher concentrations of the biomarker (results not shown). From these results, it can be concluded that the use of whole serum has not compromised the activity of both the HER-2 antigen and the AuNPs, as the assay has shown a small decrease in sensitivity as compared to the assay performed with AuNPs in buffer. Similar to the assay conducted in serum using the anti-goat Ab-HRP instead of the AuNP-Ab-HRP conjugate, a reduction in the sensor detection range was observed, again probably due to a partial blocking of the capture Ab sites by serum components. Nevertheless, the electrochemical immunosensor is still suitable for the clinical detection of HER-2 biomarkers both in healthy individuals (HER-2 between 2–15 ng mL^−1^) and in women with breast cancer (HER-2 > 15 ng mL^−1^). The proposed method was shown to be novel, easy to use and can be used as a point-of-care diagnosis.

The specificity of the immunosensor developed for HER-2 detection using the AuNPs-Ab-HRP conjugate was assessed both in buffer and undiluted serum testing the HER-1 biomarker rather than HER-2. The assay was performed with the optimized conditions testing 75 ng mL^−1^ of HER-1 in both buffers and 100% serum. For a proper comparison, the immunosensor was also tested with 25 ng mL^−1^ of HER-2 prepared both in buffer and spiked into 100% serum. Figure 7c shows that HER-1 antigen in both buffers and in 100% serum had a very low interference and was close to the response for the control (no biomarker). Moreover, it is possible to clearly distinguish between 25 ng mL^−1^ of HER-2 and 75 ng mL^−1^ of HER-1 tested. This proves the specificity of the assay for HER-2, which uses a monoclonal antibody as the capture antibody. Moreover, this proves that the structures of HER-1 and HER-2 are different making them independent from each other [10,51]. Therefore, the electrochemical sensor was considered specific to HER-2.

## 4. Discussion

Table 1 and Table 2 show a comparison of the performance of the immunosensors developed in this work with other biosensing systems reported in the literature. 

In general, the two Tables show that the LODs achieved in this work for HER-1 (30 pg mL^−1^, Table 1) and HER 2 (30–50 pg mL^−1^, Table 2) both in buffer and in serum when using gold nanoparticles for amplifying the signal are more sensitive or as good as other biosensing methods reported in the literature that also have used nanomaterials for signal amplification. Even though these methods seem novel and have low LODs, often they still require expensive equipment unlike the method proposed in this work, which in addition to being novel for the two biomarkers, is also easy to use and has the potential to be utilized as point-of-care diagnostics.

## 5. Conclusions

The development of electrochemical immunosensors for HER-1 and HER-2 quantification for breast cancer patients has been reported in this study. This approach uses a simple adsorption method to immobilize a capture antibody in an indirect ELISA sandwich format. For HER-1, this is the first report demonstrating a sensitive indirect ELISA format immunosensor with LOD and LOQ of 1.06 ng mL^−1^ and 2.1 ng mL^−1^ in buffer and 1.2 ng mL^−1^ and 1.5 ng mL^−1^ in 100% serum. The linear detection range of 0.5–75 ng mL^−1^ and the LOD of the ‘normal’ assay in both buffer and serum fall within the clinical range of HER-1 in breast cancer and healthy patients. Furthermore, the use of AuNP to amplify and increase the sensitivity of the immunosensor has proven to be successful as the detection range was reduced to 0.05–0.5 ng mL^−1^ and the LOD and the LOQ were further reduced to 30 pg mL^−1^ and 98 pg mL^−1^. This study highlights the great potential of the developed sensor to be used for HER-1 detection in breast cancer patients.

Similarly, the HER-2 assay demonstrates a sensitive indirect ELISA immunosensor with a LOD and a LOQ of 0.95 ng mL^−1^ 1.5 ng mL^−1^ in buffer and 1.47 ng mL^−1^ and 2.1 ng mL^−1^ in 100% serum. Similar to the HER-1 immunosensor, also for HER-2, the linear detection range of the normal assay (5–200 ng mL^−1^) and serum assay (5–80 ng mL^−1^) as well as their LODs fall within the clinical detection range of HER-2 in breast cancer and healthy patients. Additionally, for HER-2 the use of AuNP to amplify and increase the sensitivity of the immunosensor was successful as the detection range was reduced to 0.25–50 ng mL^−1^ and the LOD and LOQ were reduced to 30 pg mL^−1^ and 35 pg mL^−1^ for the buffer assay. Similar experimental work was conducted with 100% serum and it was found that the detection range was reduced further between 0.25 and 25 ng mL^−1^, and the LOD and the LOQ increased slightly to 50 pg mL^−1^ and 80 pg mL^−1^, respectively. However, both the linear detection range and LODs in buffer and in serum are still within the clinical range, and therefore, able to distinguish between breast cancer and healthy patients.

This study highlights the potential of the developed immunosensors to be used for HER-1 (EGFR) and HER-2 quantification, and therefore, for early detection of breast cancer in patients. Although the testing time is currently around 3 h (1 h incubation for antigen, 1 h for the detection Ab, and 1 h for the secondary IgG-HRP or AuNP-Ab-HRP conjugate) no attempts were made in this study to shorten the assay time. We are confident that these can be reduced significantly with further development without performance deterioration. Therefore, the promising results of the proposed biosensing approach could promote the future design and development of a sensitive point-of-care immunosensor integrated with a microfluidic system for the quantification of the two biomarkers in complex biological samples. This would enable both early detection of breast cancer and the monitoring of the disease’s treatment and progression with a beneficial impact on patient survival and clinical outcomes.

## Figures and Tables

**Table 1:** Comparison of the performance of the sensor developed in this work for HER-1 (EGFR) with others reported in the literature.

Assay Method	Technique	Sample	Dynamic Range	LOD	Ref.
Label-free gravimetry Anti HER-1 Ab Immobilized on a gold QCM	QCM	Buffer	0.01–10 μg mL^−1^	10 ng mL^−1^	[52]
EIS using HER-1 peptide ligand.	EIS	Buffer and serum	1 ng mL^−1^–1 × 10^−4^ ng mL^−1^	3.7 × 10^−4^ ng mL^−1^	[11]
Voltammetry of Antibody immobilized on a SAM modified gold electrode	Voltammetry	Buffer	1 pg mL^−1^–100 ng mL^−1^	1 pg mL^−1^	[26]
Forster Resonance Energy Transfer (FRET) Quantum Dots based FRET Immunoassay.	Forster Resonance Energy Transfer (FRET)	Buffer and serum	-	23 ng mL^−1^	[53]
Electrochemical immunosensor using gold SPE detecting HER-1 in buffer using an indirect ELISA format.	Amperometry	Buffer	0.5–75 ng mL^−1^	1.06 ng mL^−1^	This work
Electrochemical immunosensor using gold SPE detecting HER-1 in serum using an indirect ELISA format.	Amperometry	Serum	0.5–75 ng mL^−1^	1.2 ng mL^−1^	This work
Capacitive sensor using AuNPs for signal enhancement	Capacitive	Buffer and serum	20–1000 pg mL^−1^	20 pg mL^−1^	[54]
Anodic stripping differential pulse voltammetry Aptamer/Antibody sandwich immunoassay using gold nanoparticles	Anodic stripping differential pulse voltammetry	Buffer and serum	1–40 ng mL^−1^	50 pg mL^−1^	[30]
Photonic crystal fiber based Surface enhanced Raman scattering by using AuNP-conjugated antibody	Surface Enhance Raman Scattering	Buffer and cell lysate	-	5 ng mL^−1^	[55]
Electrochemical immunosensor using gold SPE detecting HER-1 using AuNP in an indirect ELISA format.	Amperometry	Buffer	0.05–0.5 ng mL^−1^	30 pg mL^−1^	This work

**Table 2:** Comparison of the performance of the sensor developed in this work for HER-2 with others reported in the literature.

Assay Method	Technique	Samples	Dynamic Range	LOD	Ref.
A label-free immunosensor fabricated by immobilized anti-HER-2 affibody bioreceptor on a gold nanostructure SPE.	EIS	Serum and buffer	0–40,000 ng mL^−1^	6000 ng mL^−1^	[31]
A sandwich nano-immunoassay using HER-2 nanobodies and HRP as an enzymatic label linked to a carbon SPE	Amperometry	Cell lysates and buffer	1000–200,000 ng mL^−1^	1000 ng mL^−1^	[34]
Sandwich-magnetoimmunoassay onto Protein A magnetic beads and alkaline phosphatase as an enzymatic label.	DPV	Serum and buffer	6–15 ng mL^−1^	6 ng mL^−1^	[56]
A label free immunosensor fabricated by immobilising anti-HER-2 antibody via two linkers neutravidin and biotinylated protein–A onto a gold surface via flow injection analysis.	Surface Acoustic Wave	Buffer	13–20 ng mL^−1^	10 ng mL^−1^	[22]
Nanostructures sandwich immunosensor on nano-electrode ensembles prepared in track-etch polycarbonate membranes.	CV	Cell lysates, tumour and buffer	-	40 ng mL^−1^	[27]
An indirect sandwich ELISA electrochemical immunosensor using gold SPE detecting HER-2 using an indirect ELISA format.	Amperometry	Buffer	5–200 ng mL^−1^	0.95 ng mL^−1^	This work
An indirect sandwich ELISA electrochemical immunosensor using gold SPE and serum detecting HER-2 using an indirect ELISA format.	Amperometry	Serum	5–80 ng mL^−1^	1.47 ng mL^−1^	This work
A sandwich type label free immunosensor fabricated by immobilization of anti-HER-2 using gold nanoparticle for signal enhancement.	SPR	Serum and cell lysate	0.23–55 ng mL^−1^	180 pg mL^−1^	[21]
An immunosensor fabricated by immobilization of anti-HER 2 antibody modified using multiwall carbon nanotube and gold nanoparticle in a carbon ionic liquid electrode.	EIS	Serum	10–110 ng mL^−1^	7.4 ng mL^−1^	[32]
Sandwich magneto-immunosensor onto carboxylic acid modified magnetic beads and HRP as enzymatic label.	Amperometry	Buffer	0.1–32 ng mL^−1^	26 pg mL^−1^	[57]
A label-free immunosensor fabricated by immobilization of anti-HER-2 modified Iron oxide nanoparticles onto a gold electrode.	DPV	Buffer	0.01–10 ng mL^−1^ 1–100 ng mL^−1^	0.995 pg mL^−1^	[58]
Sandwich immunoassay on a screen-printed gold electrode modified with gold nanoparticle	Linear Sweep Voltammetry	Serum	15–100 ng mL^−1^	4.4 ng mL^−1^	[36]
Immunosensor fabricated by covalently immobilizing anti-HER-2 onto a nanocomposite layer composed of self- assembled 2,5-bis(2-thienyl)-1H-pyrrole -1-(p-benzoic acid (DPB) on AuNPs. (Hyd-AuNP-Apt Bioconjugate)	Square Wave Stripping Voltammetry	Buffer	0.1 pg mL^−1^ −100 ng mL^−1^	0.037 pg mL^−1^	[37]
An indirect sandwich immunosensor using screen-printed gold electrodes detecting HER-2 modified with gold nanoparticles.	Amperometry	Buffer	0.25–50 ng mL^−1^	30 pg mL^−1^	This work
An indirect sandwich ELISA electrochemical immunosensor using screen-printed gold electrodes and serum detecting HER-2 modified with gold nanoparticles.	Amperometry	Serum	0.25–50 ng mL^−1^	50 pg mL^−1^ (0.05 ng mL^−1^)	This work

## Data Availability

The data presented in this manuscript are available on request from the corresponsding authors.
